# Response to Fässler et al’s “Successful treatment of refractory folliculitis decalvans with apremilast”

**DOI:** 10.1016/j.jdcr.2024.10.036

**Published:** 2024-12-07

**Authors:** Charlotte Dethier, Sara Azirar, Lina Verluyten, Hugo Boonen, Martine Grosber, Jan Gutermuth

**Affiliations:** a Vrije Universiteit Brussel (VUB), Universitair Ziekenhuis Brussel (UZ Brussel), Department of Dermatology, Universitair Ziekenhuis Brussel, Brussels, Belgium; b Heilig Hartziekenhuis Mol, Department of Dematology, Mol, Belgium

**Keywords:** apremilast, folliculitis decalvans

*To the Editor:* We read with great interest the recent paper by Fässler et al regarding the successful treatment of folliculitis decalvans with apremilast.^1^ Their report highlighted the effectiveness of apremilast in a patient who had not responded to a wide range of previous treatments such as topical and systemic corticosteroids, dapsone, and adalimumab. They postulated that apremilast inhibits neutrophils, which is the main inflammatory cell type in folliculitis decalvans. In their case, a flare-up was noted upon discontinuation of the therapy.

Based on this case report, we initiated apremilast treatment in a 56-year-old male patient in our outpatient clinic, who had a 30-year history of refractory folliculitis decalvans (Fig 1). Given the long disease duration and his resistance to prior therapies, an off-label treatment with apremilast was initiated, motivated by the highly positive outcomes reported by Fässler et al. Furthermore, apremilast is known to have a good safety profile.

**Fig 1:**
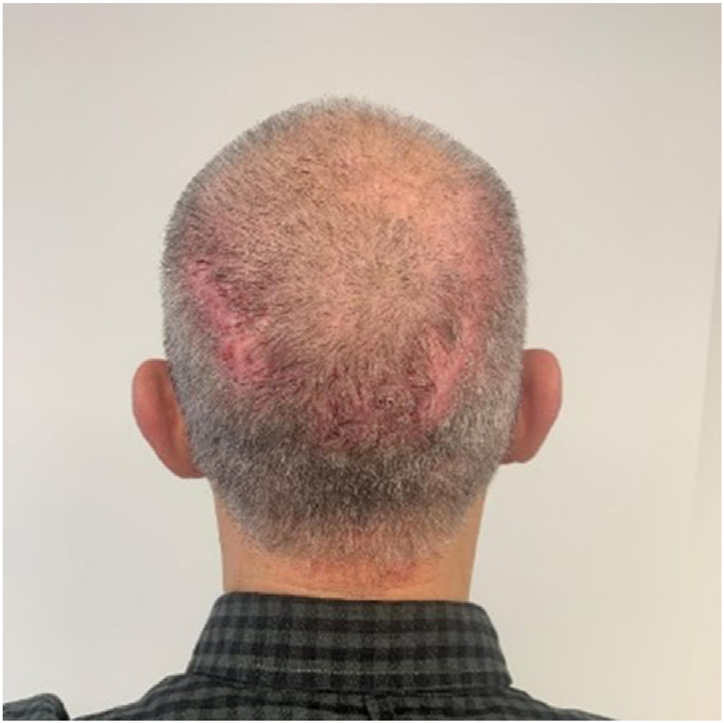
Folliculitis decalvans, before treatment with apremilast.

In our case, there was significant clinical improvement, with reduced erythema, pustules, and pruritus by the second month. After 7 months, complete remission was achieved. Importantly, after discontinuation of apremilast at 18 months, no recurrence was observed up until 26 months later (Fig 2). This outcome stands in contrast to the case described by Fässler et al, in which a flare-up occurred upon therapy discontinuation.

**Fig 2:**
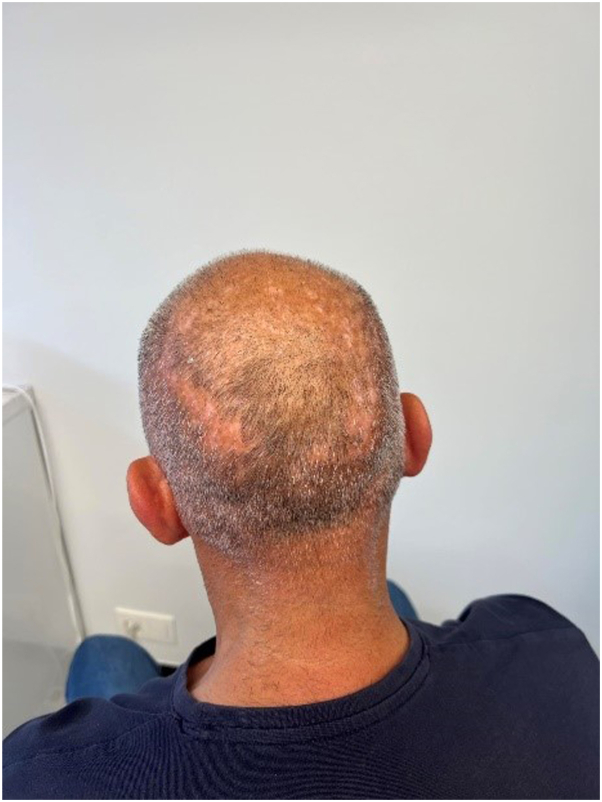
Folliculitis decalvans, 22 months after stop of treatment with apremilast.

Our findings provide additional support for the use of apremilast in treating folliculitis decalvans and align with the results of Fässler et al. Our patient’s sustained remission after cessation of therapy suggests a potential disease-modifying effect of apremilast. This report strengthens the evidence supporting apremilast as a valuable therapeutic option in this challenging condition.

## Conflicts of interest

None disclosed.
